# Research progress of calcium carbonate nanomaterials in cancer therapy: challenge and opportunity

**DOI:** 10.3389/fbioe.2023.1266888

**Published:** 2023-09-21

**Authors:** Tiantian Liang, Zongqi Feng, Xiao Zhang, Tianfang Li, Tingyu Yang, Lan Yu

**Affiliations:** ^1^ Graduate School, Inner Mongolia Medical University, Hohhot, Inner Mongolia, China; ^2^ Clinical Medical Research Center, Inner Mongolia People’s Hospital, Hohhot, Inner Mongolia, China; ^3^ Inner Mongolia Key Laboratory of Gene Regulation of the Metabolic Disease, Inner Mongolia People’s Hospital, Hohhot, Inner Mongolia, China; ^4^ Inner Mongolia Academy of Medical Sciences, Inner Mongolia People’s Hospital, Hohhot, Inner Mongolia, China

**Keywords:** calcium carbonate nanomaterials, nanotechnology, drug carrier, pH-responsive, cancer treatment

## Abstract

Cancer has keeping the main threat to the health of human being. Its overall survival rate has shown rare substantial progress in spite of the improving diagnostic and treatment techniques for cancer in recent years. Indeed, such classic strategies for malignant tumor as surgery, radiation and chemotherapy have been developed and bring more hope to the patients, but still been accompanied by certain limitations, which include the challenge of managing large wound sizes, systemic toxic side effects, and harmful to the healthy tissues caused by imprecise alignment with tumors in radiotherapy. Furthermore, immunotherapy exhibits a limited therapeutic effect in advanced tumors which is reported only up to 25%–30%. The combination of nanomaterials and cancer treatment offers new hope for cancer patients, demonstrating strong potential in the field of medical research. Among the extensively utilized nanomaterials, calcium carbonate nanomaterials (CCNM) exhibit a broad spectrum of biomedical applications due to their abundant availability, cost-effectiveness, and exceptional safety profile. CCNM have the potential to elevate intracellular Ca^2+^ levels in tumor cells, trigger the mitochondrial damage and ultimately lead to tumor cell death. Moreover, compared with other types of nanomaterials, CCNM exhibit remarkable advantages as delivery systems owing to their high loading capacity, biocompatibility and biodegradability. The purpose of this review is to provide an overview of CCNM synthesis, focusing on summarizing its diverse roles in cancer treatment and the benefits and challenges associated with CCNM in cancer therapy. Hoping to present the significance of CCNM as for the clinical application, and summarize information for the design of CCNM and other types of nanomaterials in the future.

## 1 Introduction

Cancer is the second leading cause of mortality worldwide (Sung et al., 2021). According to data of 2020, there were 19.29 million new cancer cases worldwide, including 10.6 million males and 9.23 million females. Respectively, 5.53 million males and 4.43 million females die from cancer worldwide (Xia et al., 2022). Consequently, cancer has imposed a substantial economic burden on global healthcare systems. Surgery, chemotherapy and radiotherapy have been the classical cancer treatment. Surgery is the most efficient way to remove the solid tumor, however, incomplete resection and the possibility of helping the cancer cell metastasis during resection are the unavoidable problems (Curtis et al., 2020; Yamaguchi et al., 2021). Chemotherapy and radiotherapy bring fatal damage not only to the cancer cells, but the normal cells, let alone the drug resistance and the immunosuppression which is caused by the bone marrow damage (Behranvand et al., 2022). Furthermore, radiotherapy itself could cause cell unknown mutations and the metastasis (Ruysscher et al., 2019). In recent years, the emerging immunotherapy has received widespread attention due to its reasonable theory and the optimistic effects; whereas, the percentage of the patients who could benefit from it is comparatively low because of the significant heterogeneity of cancer (Liu et al., 2021). Nanomaterials have played an active role in the biomedical field, especially in cancer treatment (Kashyap et al., 2023; Pei et al., 2023). The inherent characteristics of the nanomaterial, such as particle size, atomic composition, magnetic and electronic specificity confer the nanoparticles with unparalleled advantages in treating diseases, particularly in targeting therapy for cancer (Wang et al., 2023a). By modifying nanoparticles in physical, chemical, and biological activity aspects, their dissolution is promoted, absorption is improved, and delivery efficiency is enhanced (Zhu and Li, 2023). The use of nanoparticles to control the delivery and release of anticancer drugs has become a hot topic in the research of nanocarrier drugs (Yang et al., 2022). Among numerous nanomaterials, calcium carbonate nanomaterials (CCNM) have distinctive properties: good biocompatibility, easily synthesized, and could be produced in diverse forms and crystal structures (Luo et al., 2020; Li et al., 2021a).

**FIGURE 1:**
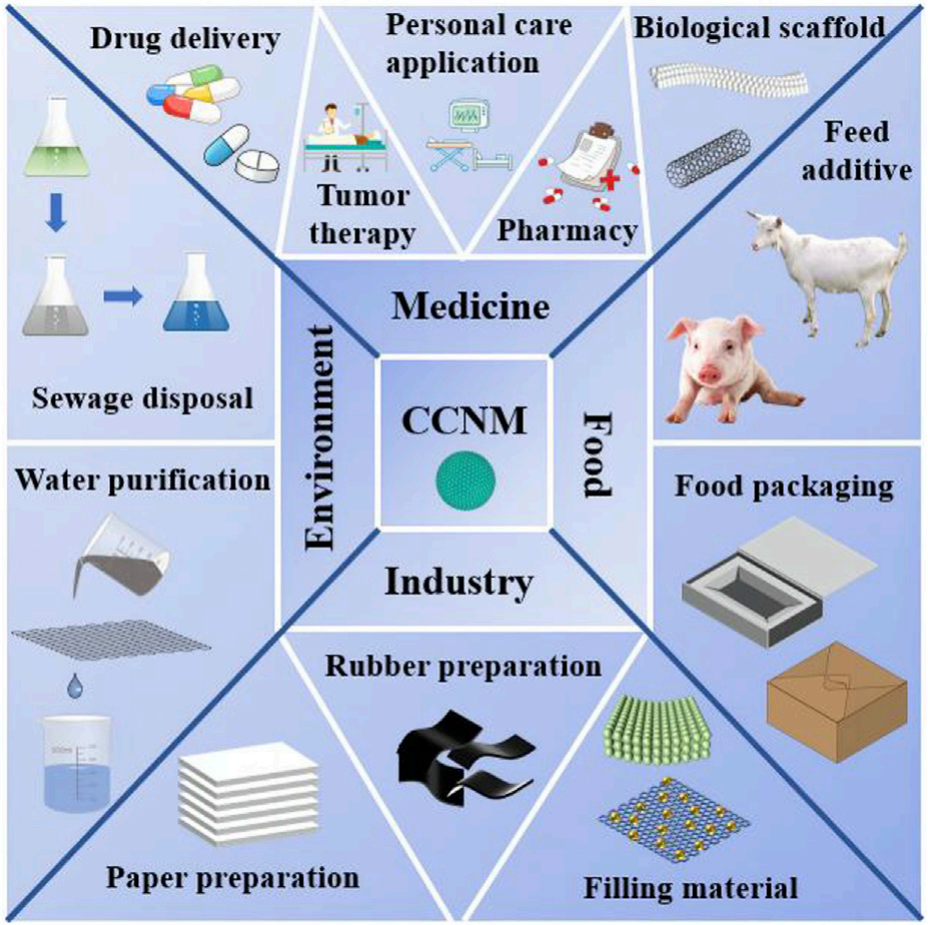
Applications of CCNM in different fields.

CCNM are widely used in various industries such as food packaging, pharmaceuticals, paint pigments, and polymer fillers (Shou et al., 2022). Meanwhile, it shows great potential in biomedicine, environmental remediation and energy production, etc. (Figure 1). In biomedicine field, CCNM can be used in treating cancer, disease detection and bone regeneration (Vikulina et al., 2021). Due to the biocompatibility and high specific surface area of CCNM, they possess the remarkable advantages in drug delivery (Mao et al., 2021). CCNM could provide Ca^2+^, attack mitochondria, and further kill malignant cells in cancer therapy (Bai et al., 2022).

In this review, we first reviewed the main synthesis methods of CCNM, followed by summarizing their different functions in cancer treatment, including drug carriers, synergistic therapy, Ca^2+^ overload therapy, etc. Finally, we delineated the merits and challenges of CCNM in the context of cancer therapy. Hoping to present the significance of CCNM as for the clinical application, and provide some clues for the novel design of CCNM and other types of nanomaterials in the future.

## 2 Synthesis of CCNM

There are currently various methods for synthesizing CCNM. According to different synthesis techniques, various shapes and sizes of particles can be obtained (Wu et al., 2022). All of the synthesis parameters, including reactant concentration, stirring strength of the reaction mixture, temperature, and solvent type, affect the crystal formation, particle size, and stability of CCNM (Li et al., 2020a; Devi et al., 2023). The main synthesis methods of CCNM, such as precipitation method (Febrida et al., 2021), mechano-chemical method (Preksha et al., 2021), sea shell and eggshell mediated (Azraian and Sutapun, 2022) and gas diffusion method (Gindele et al., 2021) are summarized as follows Table 1.

**TABLE 1:** Comparison of advantages and disadvantages of common synthesis methods of CCNM.

Synthetic method	Scope of application	Superiority	Drawback	(Refs.)
precipitation method	laboratory	low cost, simple and easy to operate	uneven size and morphology	Febrida et al. (2021)
mechano-chemical method	environment, biomedical	difficult to reunite	easy to be contaminated and instrument complexity	Preksha et al. (2021)
sea shell and eggshell mediated	biomedicine	rich source, non-toxic, green, with drug loading capacity	impurity of nanoparticles	Azraian and Sutapun (2022)
gas diffusion method	biomedicine	low cost, easy to obtain	easy to agglomerate	Gindele et al. (2021)

### 2.1 Precipitation method

Spontaneous precipitation reaction is that the mixture of calcium and supersaturation solution of carbonate are simply blended. It is the most important and simplest method to prepare CCNM (Sun et al., 2020). Persano et al. (2022) synthesized CCNM using this method and studied in detail the effects of different synthesis parameters. They found that as the stirring speed increased, the nano size of CaCO_3_ decreased correspondingly. Meanwhile, it was found that the higher the salt concentration, the higher the supersaturation became, and thus the smaller the nano size. Yang et al. (2021a) used CaCl_2_ and NaCO_3_ as raw materials, injected NaCO_3_ into the CaCl_2_ containing Ca(OH)_2_ to prepare CCNM, and studied the use of Ca(OH)_2_ as an additive. As a non-impurity additive, Ca(OH)_2_ increased the pH value of the suspension. The results showed that the higher the pH value of the reaction system, the smaller the CCNM size. Pérez-Villarejo et al. (2018) reported a simple and rapid method for synthesizing CCNM. The experiment used Ca(NO_3_)_2_·4H_2_O and NaHCO_3_ as raw materials and mainly observed the role of sucrose as an additive in the synthesis of CCNM. Their conclusion is that sucrose interacts with Ca^2+^ and water molecules through hydroxyl groups. The higher the concentration of sucrose, the more crystalline forms of synthetic vaterite, and the smaller the size of nanoparticles. Sucrose can stabilize the vaterite crystal form, making it difficult to transform into a more stable calcite crystal form, which provides a fast and simple method for the synthesis of vaterite crystal form in the future.

Based on the application experience, the precipitation method has been reported to involve the addition of different molecules, including synthetic polymers, surfactants and biomolecules, to synthesize CCNM of different sizes and shapes. This method can adjust the crystal morphology and particle size of CCNM by changing the concentration of reactants and adding organic matter. However, the rapid reaction speed of precipitation reaction, which is not conducive to observing and studying the reaction process. This might be its main drawback.

### 2.2 Mechano-chemical method

The mechano-chemical synthesis method to produce CCNM is the process involving mechanical fragmentation and chemical reactions (Cestari et al., 2021). Its chemical reaction occurs through the absorption of mechanical energy by reactants. This method is divided into the dry mechano-chemical and the wet mechano-chemical method (Mkhize et al., 2021). Gbadenyan et al. (2021) prepared CCNM by mechano-chemical grinding method. They started with using stainless steel balls and cans to grind the snail shell to obtain fine particles. Then, the fine particles are placed in a stainless steel wide-mouthed bottle, solvent is added, and wet grinding is performed to obtain CCNM. Compared with precipitation method, the equipment setup for the mechano-chemical method is cost-effective and enables the agglomeration-free production of nanoparticles with narrow particle size distribution (Piras et al., 2019). But this method also has some drawbacks, such as instrument rusting, which may hinder the synthesis process of nanoparticles and cause certain pollution.

### 2.3 Sea shell and eggshell mediated

It is advantageous to synthesize CCNM using natural reserves of CaCO_3_, such as eggshells and seashells, as it is easy to obtain, non-toxic, and biocompatible, making it an ideal candidate for biomedical applications (Prihanto et al., 2023). Rinu et al. (2020) synthesized nanoparticles using eggshells as raw materials. By collecting a large amount of eggshells and drying them at room temperature. Then gently crush the eggshell and place it in a crucible, keeping it in a muffle furnace at 900°C for 2 h. Finally, it is turned into fine powder to synthesize CCNM. Khan et al. (2019) used eggshells and agar as raw materials to calcine the synthesized products at high temperatures, ultimately synthesizing CCNM. The calcination temperature has a significant impact on the morphology, composition, and size of particles. Hussein et al. (2020) crushed the shells into powder and mixed them with HCl to form CaCl_2_. And the effect of different volumes of double-distilled water (DDW) on the morphology of CCNM derived from shells was studied. Nanoparticles prepared with different volumes of DDW have different shapes and sizes, which is due to the dilution of the solution volume by DDW, resulting in changes in the activity space of the nanoparticles.

Seashells and eggshells are abundant in resources, inexpensive, and easy to obtain, which can reduce environmental pollution levels and have good economic and social benefits. From this, it can be seen that CCNM obtained through shells and eggshells has enormous advantages, but the resulting nanoparticles are impure, which requires further exploration.

### 2.4 Gas diffusion method

The gas diffusion method has been widely used to synthesize CCNM in the biomedical field. This method involves the thermal decomposition of (NH_4_)_2_CO_3_ or NH_4_HCO_3_ to generate CO_2_ and NH_3_ diffusion into an ethanol solution containing calcium salts to generate precipitates or the introduction of CO_2_ gas into the calcium salt ethanol solution at atmospheric pressure to synthesize CCNM (Wang et al., 2020). Chuzeville et al. (2022) used this method to synthesize the dispersed CCNM below 150 nm in ethanol. Ethanol is the main solvent to ultimately obtain stable and uniform spherical nanoparticles, which can inhibit the spontaneous aggregation and crystallization of CCNM. Ju et al. (2022) made some improvements on the basis of the original steps. In the ethanol-water binary system, stable amorphous calcium carbonate (ACC) nanospheres were synthesized by gas diffusion method. And different volumes of ammonia were added to the solution, it was observed that as ammonia was continuously added, the volume of the nanomaterials continued to decrease. Xu et al. (2022a) synthesized ACC by gas diffusion method, added Ca(OH)_2_ and CO_2_ in the methanol-water system, reacted in an autoclave and centrifuged to obtain ACC. The research results indicate that with the increase of water content in the solution system, ACC gradually changed into a metastable vaterite crystal.

Compared to other synthesis methods, gas-phase diffusion method has the advantage of controllable reaction speed. There is no need for other additives, and higher product quality could be acquired. Thus, it is often used as the preferred method for studying the biomimetic synthesis of CCNM minerals. But the obtained nanoparticles are still easy to agglomerate. Polyethylene glycol and other substances can be used to modify the surface of CCNM to improve the dispersion and stability in aqueous solution.

## 3 Application of CCNM in cancer treatment

**FIGURE 2:**
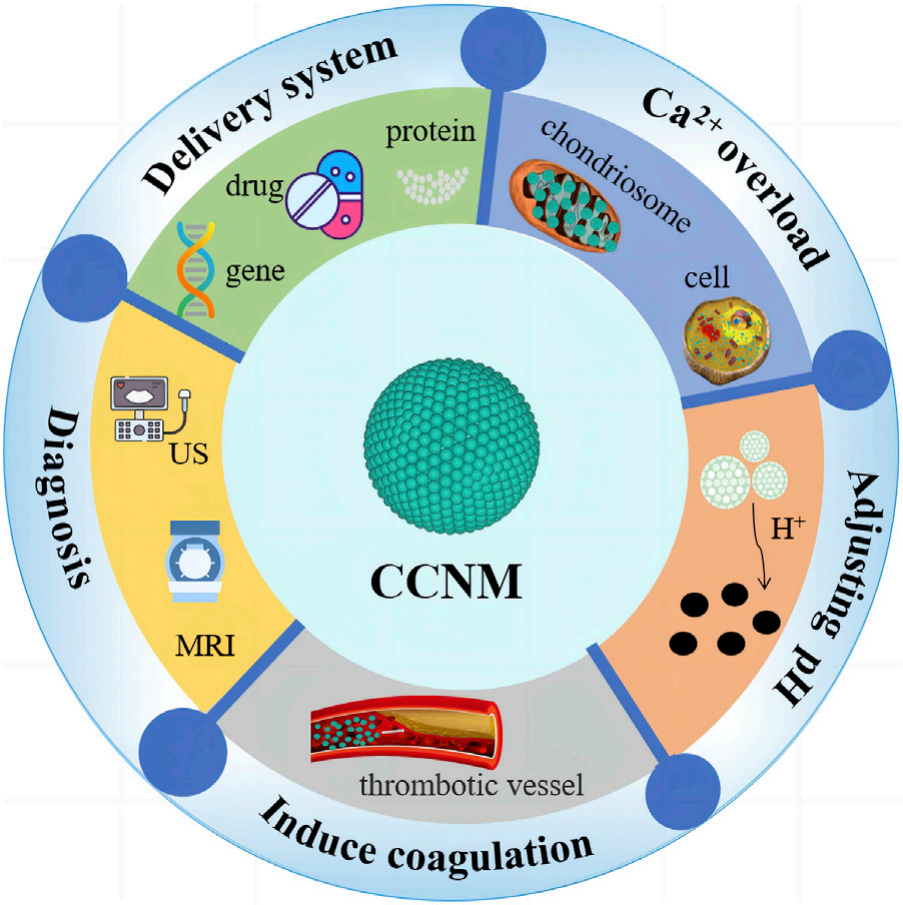
Applications of CCNM in cancer treatment.

CCNM not only have great potential in imaging and biosensing but also play an important role in fields such as dental materials and bone regeneration (Li et al., 2021b; Niu et al., 2022). Due to their excellent biocompatibility and pH responsiveness, CCNM have great developing prospects in cancer treatment, especially in delivery systems, tumor diagnosis, Ca^2+^ overload, pH regulation, and coagulation induction (Novoselova et al., 2021; Zhao et al., 2023) (Figure 2). The different roles of CCNM in cancer treatment are listed in Table 2.

**TABLE 2:** Different roles of CCNM in cancer treatment.

Composition	Preparation method	Therapeutic methods	CCNM function	(Refs.)	
CUR, CCNM	gas diffusion	Ca^2+^ overload/immunotherapy	carrier, Ca^2+^ overload		Zheng et al. (2022)
DOX, CCNM	coprecipitation	chemotherapy	carrier		Popova et al. (2021)
ICG, DOX, CCNM	gas diffusion	chemotherapy/PTT	carrier		Yu et al. (2023)
hAS, PDA, PEG, CCNM	gas diffusion	PTT/chemotherapy	carrier		Zhong et al. (2022)
Cu_2_O, HA, CCNM	gas diffusion	PTT/photodynamic therapy (PDT)/chemodynamic therapy (CDT)/Ca^2+^ overload	Ca2+ overload		Chang et al. (2020)
CDDP, OA, CCNM	micro emulsion	chemotherapy	carrier		Khan et al. (2022)
Capsaicin, CCNM	gas diffusion	Ca^2+^ overload	carrier, Ca^2+^ overload		Xu et al. (2022b)
Ce6, Cu^2+^, CCNM	gas diffusion	Ca^2+^ overload/CDT/sonodynamic therapy (SDT)	Ca^2+^ overload		Zhao et al. (2022a)
TCL, CpG, CCNM	precipitation	immunotherapy	consuming excessive hydrogen ions and lactate, carrier		Ding et al. (2023)
Fe^2+^, GA, Pt (IV)-SA, CCNM	precipitation	ferroptosis/chemotherapy	carrier		Han et al. (2022)
iridium (III), CCNM	gas diffusion	Ca^2+^ overload/PDT	carrier, Ca^2+^ overload		Shen et al. (2022)
Fe^2+^, CCNM, O_2_, COF, FA	gas diffusion	PDT/Ca^2+^ overload	Ca^2+^ overload		Zhou et al. (2022)
KAE, M, CCNM	gas diffusion	Ca^2+^ overload/chemotherapy	Ca^2+^ overload		Li et al. (2021c)
CDDP, CUR, CCNM, PEG	gas diffusion	Ca^2+^ overload/chemotherapy	Ca^2+^ overload		Zheng et al. (2021)
CCNM	gas diffusion/double decomposition reaction	alkalization of TME	modulate tumor pH		Som et al. (2016)
CCNM	gas diffusion	alkalization of TME	modulate tumor pH		Lam et al. (2021)
DOX, CCNM	emulsion	starving tumor therapy/chemotherapy	induce blood coagulation, carrier		Li et al. (2020b)
NaGdF_4_, CCNM	gas diffusion	___	carrier, produce CO_2_ aid in imaging		Yi et al. (2019)
DOX, CCNM	*in-situ* polymerization	chemotherapy	carrier, produce CO_2_ aid in imaging		Huang et al. (2020)

### 3.1 CCNM as carriers for delivering anticancer drugs

An ideal anticancer drug delivery system is to deliver the drug directly to the target site, with minimal impact on normal cells, thereby improving treatment efficiency and producing minimal toxic side effects. Numerous tumor targeted drug delivery systems have been constructed through nanotechnology, which not only improves drug stability but also limits drug toxicity (Elbaz et al., 2020). CCNM have received great attention among different inorganic nanocarriers. Their natural characteristics such as biocompatibility, pH responsiveness and high encapsulation efficiency make them an ideal carrier for transporting various bioactive substances, especially anticancer drugs and genes (Fu et al., 2019). Therefore, when CCNM are coupled with drugs and genes, they improve the efficiency of disease treatment.

At present, there are two main ways for CCNM to deliver drugs for tumor treatment. One method involves directly binding anti-tumor drugs onto the surface of CCNM, while the other method involves co-doping drugs with CCNM and allowing the complex to enter tumor cells (Teixeira et al., 2022). Zheng et al. (2022) used the one-pot gas diffusion method to co-dope curcumin (CUR) with CCNM, and the obtained CCNM can decompose at low pH to release CUR. Among them, CUR causes cell apoptosis by affecting Ca^2+^ homeostasis. By using CCNM as a carrier to transport CUR, the programmed release of CUR at the tumor site was achieved, improving the transport efficiency and anticancer activity of CUR.

Popova et al. (2021) loaded DOX onto the surface of CCNM, with the aim of studying the coupling efficiency of CCNM with drugs and the effectiveness of carrying DOX in inhibiting cancer cell proliferation *in vitro*. In an *in vitro* model, the composite material of CCNM and DOX was shown to effectively inhibit the growth of cancer cells. The characteristics of CCNM also help prevent unnecessary accumulation of DOX in major organs such as the liver, heart, and kidneys.

**FIGURE 3:**
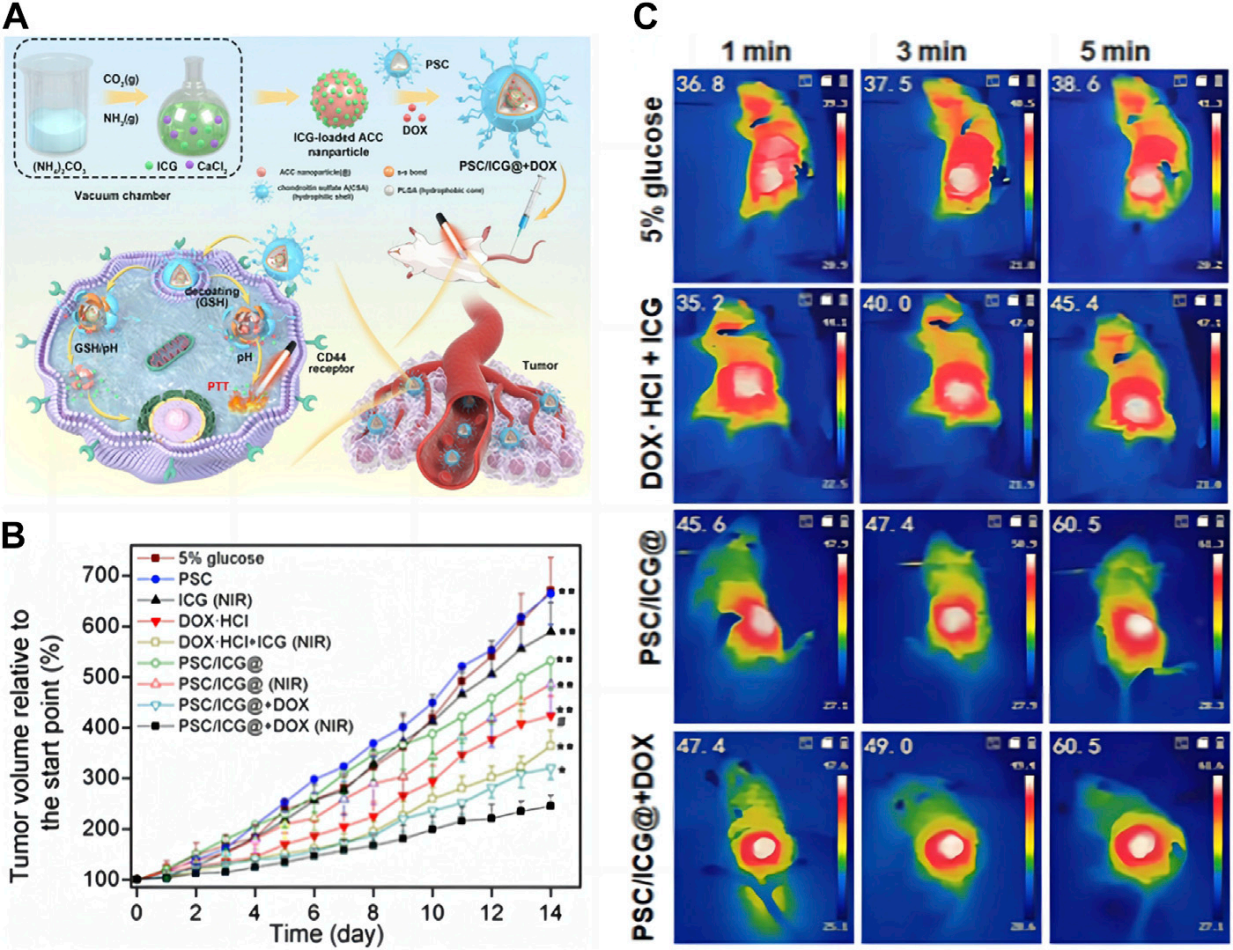
CCNM are loaded with active substances to treat tumors: **(A)** Schematic diagram of PSC/ICG@+DOX nanoparticle synthesis and mediated apoptosis; **(B)** Tumor growth curves of mice after the treatment; **(C)** Laser imaging of mice (Yu et al., 2023). Copyright 2023 Dove Medical Press Limited.

On this basis, drug delivery systems can be designed to combine multiple treatment methods into an intelligent carrier, providing a solution for their potential applications in cancer diagnosis and treatment. As shown in Figure 3A, Yu et al. (2023) co-doped CCNM with indocyanine green (IGG) before loading DOX on the surface, then encapsulated them with poly (lactic-co-glycolic acid)-ss-chondroitin sulfate A (PSC). This study combines photothermal therapy (PTT) with chemotherapy to treat tumors. PTT can use photosensitizers to generate heat energy and ultimately eliminate tumor cells. IGG is an effective photosensitizer, but free IGG cannot selectively reach the tumor site. Utilizing CCNM delivered IGG to reach the tumor site for PTT. This method not only enables IGG to accurately reach tumor cells but also significantly increases the metabolic time of IGG and improves treatment efficiency. As shown in Figure 3B, compared with other control groups, the experimental group significantly inhibited the tumor volume of mice. It was observed in the mouse thermal image in Figure 3C that this nanomaterial helps IGG to better exert its therapeutic effect. Observing from the picture, the tumor temperature in the 5% glucose group was slightly increased after 5 min of laser irradiation. The free DOX + ICG group exhibited the tumor temperature with an increase to 45.4°C. Notably, PSC/ICG@ and PSC/ICG@+DOX groups showed an increased temperature of 60.5°C after 5 min irradiation, which could effectively generate hyperthermia for PTT of malignant tumors. From this, it can be seen that CCNM have enormous advantages in delivery systems. At the same time, we can further study its synthesis method and functionalization to generate CCNM with different morphologies, thereby achieving higher loading efficiency and achieving better therapeutic effects.

### 3.2 Ca^2+^ overload

As a pivotal second messenger in cellular signaling, Ca^2+^ orchestrates the precise regulation of diverse physiological processes through targeted activation of specific proteins (Liu et al., 2020c). Under normal circumstances, the concentration of cytoplasmic free Ca^2+^ is maintained at a significantly low level of approximately 100 nmol/L. There is a 10^4^ ∼ 10^5^ -fold gradient of Ca^2+^ concentration between outside and inside of cells (Bai et al., 2022). Without the stimulation of abnormal conditions, the intracellular Ca^2+^ concentration is lower than the level of extracellular fluid, which plays an active role in regulating cells (Ma et al., 2020; Eisner et al., 2023). But if the concentration of Ca^2+^ exceeds the normal range, it will cause damage to the cells. Therefore, regulating Ca^2+^ concentration has broad research prospects in tumor treatment. In recent years, Ca^2+^ overload treatment has garnered significant attention in the field of tumor treatment, with a close association between Ca^2+^ and mitochondria (Zhu et al., 2019). When intracellular Ca^2+^ overload occurs, the mitochondrial Ca^2+^ uniporter (MCU) sequesters intracellular Ca^2+^. Upon reaching a certain threshold, this uptake of Ca^2+^ by mitochondria triggers the opening of the mitochondrial permeability transition pore (PTP) (Glitsch, 2019; Delierneux et al., 2020). When PTP is opened, many large molecules non-selectively diffuse from cytoplasm to mitochondria, leading to the destruction and dysfunction of mitochondrial membrane potential. Mitochondrial Ca^2+^ overload can lead to the release of cytochrome C and the activation of aspartate proteolytic enzymes, thereby damaging the production of reactive oxygen species related to the respiratory chain and affecting mitochondrial function, ultimately leading to cell apoptosis (Calvo-Rodriguez and Bacskai, 2021). After mitochondrial damage, biological transmission electron microscopy (Bio-TEM) is commonly employed to examine the morphology and abundance of mitochondria. At the same time, Flou-4 AM calcium probe is commonly used to detect intracellular Ca^2+^ (Zheng et al., 2021), while the Rhod-2 AM probe is used for detecting Ca^2+^ in mitochondria (Wang et al., 2023b).

**FIGURE 4:**
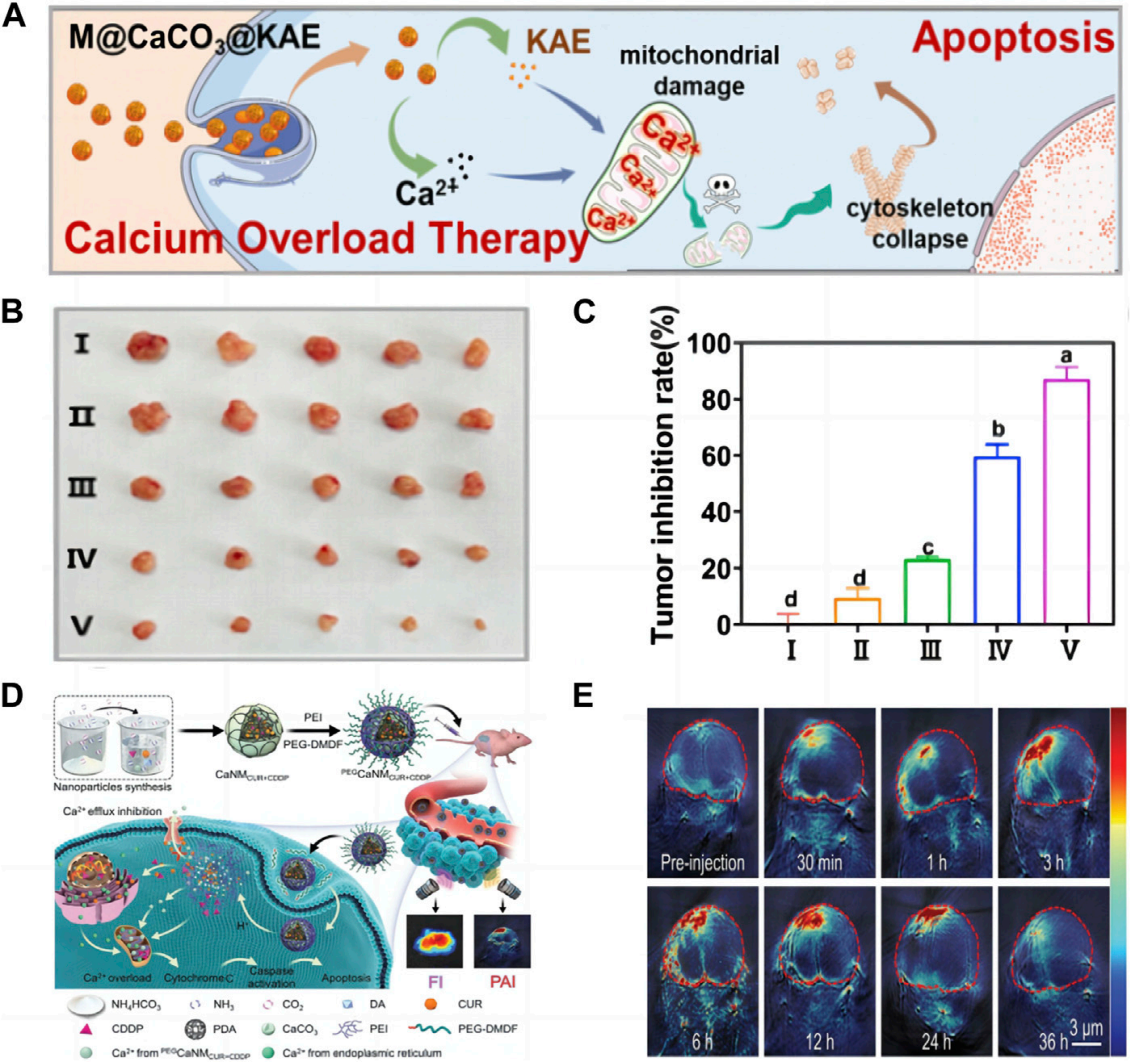
**(A)** Schematic illustration of M@CaCO_3_@KAE NP-mediated apoptosis; **(B)** The photos of tumors; **(C)** The inhibition rate of tumor weights I: Control; II: CaCO_3_ NPs; III: KAE; Ⅳ: CaCO_3_@KAE NPs; Ⅴ: M@CaCO_3_@KAE NPs (Li et al., 2021c) Copyright 2021 Elsevier; **(D)** Schematic diagram of cell apoptosis mediated by ^PEG^CaNM_CUR+CDDP_ nanoparticles; **(E)** PA imaging of mice (Zheng et al., 2021). Copyright 2021 WILEY-VCH Verlag GmbH & Co. KGaA, Weinheim.

Inadequate Ca^2+^ at the tumor site, intracellular Ca^2+^ channels can also effectively regulate the concentration of Ca^2+^, so it is difficult to achieve effective Ca^2+^ overload. Providing Ca^2+^ solely through calcium materials cannot achieve satisfactory results (Kong et al., 2021). Therefore, more and more collaborative treatment methods have been developed and have attracted widespread attention. For example, combining calcium nanomaterials with treatment methods such as chemotherapy can not only provide Ca^2+^, but also disrupt calcium homeostasis, thereby achieving more effective Ca^2+^ overload. As shown in Figure 4A, Li et al. (2021c) designed the M@CaCO_3_@KAE nano platform, thus realizing the cooperative and targeted therapy of tumors. Among them, Kaempferol-3-O-Rutinoside (KAE) can effectively disrupt the regulation of Ca^2+^ homeostasis and promote Ca^2+^ influx, while CCNM can provide Ca^2+^, ultimately achieving Ca^2+^ overload and mitochondrial structure and function are disrupted, causing cytoskeletal collapse and oxidative stress, leading to cancer cell apoptosis. In Figures 4B, C, it was observed that the tumor volume of mice was effectively suppressed, indicating that this nanomaterial can to some extent inhibit tumor growth, which is of great significance for clinical applications. This experiment cleverly combines KAE with CaCO_3_, greatly increasing the concentration of intracellular Ca^2+^, thereby achieving more efficient treatment of tumors with Ca^2+^ overload, providing a unique and novel approach for our future Ca^2+^ overload experiments. In addition, as shown in Figure 4D, Zheng et al. (2021) successfully prepared CCNM codoped with cisplatin (CDDP) and CUR. They destroyed the structure and function of mitochondria through CDDP, and destroyed the endoplasmic reticulum through CUR, so that a large amount of Ca^2+^ entered the cytoplasm, and CaCO_3_ provided a large amount of Ca^2+^, thus achieving efficient Ca^2+^ overload. Due to the fluorescence characteristics of CUR, it was observed in Figure 4E that the fluorescence signal of the mouse tumor site became stronger within the first 12 h, further indicating that the CCNM nano platform has a certain degree of targeting and can accumulate within the tumor. The Ca^2+^ nanomodulator designed in this experiment achieves Ca^2+^ overload in multiple directions, targeting cancer through mitochondria, and has great prospects in clinical practice. Ca^2+^ overload is one of the most effective methods to induce cancer cell apoptosis. Upregulation of Ca^2+^ concentration in mitochondria can lead to a series of mitochondrial diseases, including decreased mitochondrial membrane potential, decreased ATP levels, altered mitochondrial morphology, and mitochondrial respiratory disorders (Guan et al., 2020). The use of ion interference to treat tumors is extremely ingenious in concept, providing a new perspective for tumor treatment. Ca^2+^ overload has gradually become a research hotspot, but simply killing tumor cells through Ca^2+^ overload is far from enough. We can use other methods for combined therapy to greatly improve the treatment efficiency of tumors.

### 3.3 CCNM modulate tumor pH

Due to metabolic disorders in tumors, the extracellular pH of solid tumors is lower than that of normal tissues. The acidic extracellular environment of tumors enhances their invasiveness and metastasis, but there are few methods to selectively regulate the extracellular pH environment of tumors. It is impractical and non-selective to flush biological system instantaneously with alkaline liquid or proton pump inhibitor (Worsley et al., 2022). Among various methods for regulating the acidic environment of tumor cells, CCNM are particularly effective because they have efficient buffering ability and can directly regulate pH values.

On this basis, Som et al. (2016) prepare monodisperse CCNM with a size range of 20–300 nm, intentionally adjusting the pH value of the tumor microenvironment (TME). It utilized a mouse cancer model and repeated daily injections of CCNM, which significantly inhibited tumor growth. And in detecting dynamic pH values in tumor mice, it was observed that the pH value increased almost linearly during the first 30 min. This experiment demonstrates that CCNM can increase the pH value of tumors and inhibit tumor growth over time in tumors. It demonstrated for the first time the ability of using CCNM to regulate the pH value in solid tumors and observed that the effective alkalization of tumor acidic pH depends on the size of the nanoparticles. It should be noted that in order to achieve sustained tumor suppression effects in future experiments, it is necessary to optimize the dosage and use CCNM in combination with other therapies to improve the targeting of CCNM to tumors. Lam et al. (2021) co incubated cancer cells and fibroblasts and then treated them with CCNM. The results showed that nanoparticles selectively inhibited the growth of tumor cells, and reduced the cell migration of these cells, but had no effect on fibroblasts. This indicates that CCNM can limit the invasiveness of tumor cells without affecting the growth and behavior of surrounding stromal cells. The advantage of this experiment compared to other experiments is that it uses microfluidic devices to simulate the flow parameters in the body to determine the impact of pH changes on tumor survival and migration. Research has shown that CCNM can indeed alter the acidic microenvironment of tumors, but relying solely on CCNM to alter the acidic environment is still not enough.

### 3.4 CCNM induce coagulation

**FIGURE 5:**
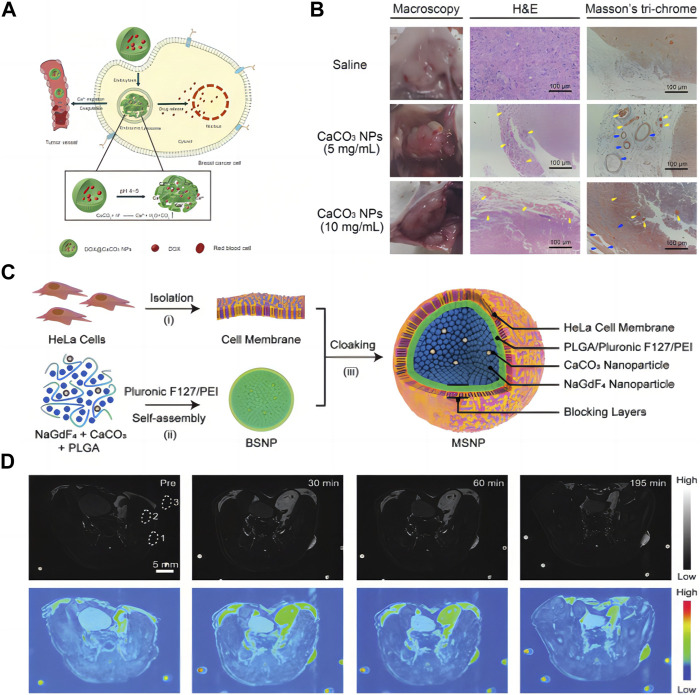
**(A)** CCNM induces blood coagulation; **(B)** Observation of blood coagulation *in vivo* (Li et al., 2020b) Copyright 2020 Royal Society of Chemistry; **(C)** Schematic diagram of synthesis of nanomaterials; **(D)** MRI of mice (Yi et al., 2019). Copyright 2019 WILEY-VCH Verlag GmbH & Co. KGaA, Weinheim.

To remedy the intrinsic deficits in energy production, cancer cells typically increase their uptake of extracellular glucose. Thus, the glucose deprivation is an effective way to cause the rapid and massive death of cancer cells, which is the essence of starving cancer cells. Based on the concept of cancer hunger treatment, as shown in Figure 5A, Li et al. (2020b) explored the effect of CCNM on coagulation. In Figure 5B, it was observed that in a mouse breast tumor model, CCNM were injected locally and physiological saline was used as the control. Through naked eye, HE, and Masson trichrome staining, it was found that CCNM, under the influence of acid stimulation, can cause coagulation reactions and thrombosis in tumor blood vessels, resulting in abnormal blood circulation and glucose supply. Therefore, CCNM have great prospects in the field of cancer treatment. Among them, Ca^2+^ activates prothrombin to thrombin, thereby causing blood to coagulate. And as the pH decreases, CCNM release more Ca^2+^, causing faster blood coagulation. The characteristic of CCNM that are pH sensitive and induce blood coagulation makes them a promising material for cancer hunger treatment. Although this study demonstrates that CCNM can induce coagulation in tumor blood vessels, it still cannot cover all tumor blood vessels, which requires further exploration.

### 3.5 Application of CCNM in tumor diagnosis

In recent years, the development of high-performance contrast agents in magnetic resonance imaging (MRI) has received great attention. As shown in Figure 5C, Yi et al. (2019) designed and synthesized a new type of nanoparticle contrast agent, including self-assembled NaGdF_4_ and CaCO_3_ nanoconjugates, providing an integrated MRI nano platform with high tumor selectivity and good biocompatibility. In this design, the spatial confinement of the Gd^3+^ leads to an “OFF” MRI signal due to insufficient interaction between the protons and the crystal lattices. However, when immersed in the mildly acidic TME the embedded CCNM generate CO_2_ bubbles and subsequently disconnect the nanoconjugates, thus resulting in an “ON” MRI signal. Due to the excellent performance of nanomaterials *in vitro*, Yi et al. (2019) investigated their comparative ability *in vivo* (Figure 5D). T1-weighted MR images were acquired *in vivo* before and after intravenous injection of nanoconjugates. The tumor site is relatively dark before injection of nanoconjugates, and it starts to light up about 30 min after injection. The contrast enhancement at the tumor site reached a tumor-to-background ratio of approximately 48 at 195 min postinjection. Compared with commonly used magnetic contrast agents, the *in vivo* performance of NaGdF_4_-CaCO_3_ shows a 60-fold enhancement in tumor visualization. It is suitable for deep tissue imaging, with higher sensitivity and selectivity, which is of great significance for constructing intelligent magnetic resonance imaging.

In addition, CCNM can generate CO_2_ bubbles under acidic conditions, which can then enhance ultrasound imaging signals. Huang et al. (2020) developed a diagnostic nanoparticle system for ultrasound and fluorescence dual-mode imaging. The nanoparticle loaded with DOX showed the ability to simultaneously treat cancer. Therefore, loading DOX onto CCNM yields CaCO_3_-DOX nanoparticles. Under acidic conditions, CCNM generate CO_2_ to enhance ultrasound imaging and increase the release of DOX, thereby improving the quality of ultrasound imaging. Also, ICG was encapsulated into CCNM, to further detect the tumor with fluorescence. Overall, this study integrates imaging and therapeutic functions to provide a promising and attractive strategy for cancer treatment. Through research, it has been found that CCNM have certain help and advantages in the diagnosis of tumors. But there are still shortcomings such as instability and weak targeting of CCNM. It is necessary to improve their targeting and stability in order to maximize the diagnostic role of CCNM in tumors. Meanwhile, Vidallon et al. (2020) synthesized hybrid CCNM with biocompatibility and pH sensitivity through rapid precipitation method. Among them, CCNM can undergo decomposition under acidic conditions to generate high echo CO_2_ bubbles, increasing the contrast intensity of ultrasound and demonstrating its potential as an ultrasound contrast agent.

Compared to traditional ultrasound contrast agents, CCNM not only achieve long-term stability of ultrasound imaging but also can quickly clear in the body without causing harm to the human body (Ma et al., 2022). At the same time, appropriate surface modification can also endow particles with specific targeting. The mesoporous structure generated by CCNM polymerization can encapsulate therapeutic drugs such as chemotherapy and sound sensitizers with good drug loading and release performance (Qiao et al., 2020). Therefore, combining the diagnosis and treatment functions of CCNM play an important role in improving the diagnosis and treatment technology of tumors.

## 4 Advantages of CCNM

Now the enormous potential of CCNM have gradually been discovered in the field of biomedicine. CCNM not only have unique characteristics such as good biocompatibility and low cost but also have a large specific surface area and are easy to functionalize on the surface (Ren et al., 2023; Torgbo et al., 2023). Therefore, CCNM play a huge advantage in delivery systems. In addition, the acid responsive properties of CCNM have great application value in passive and active targeting of cancer cells and have received widespread attention in cancer treatment, bringing new hope to cancer treatment (Wang et al., 2022).

### 4.1 Acid sensitivity

The acid sensitivity of CCNM make them particularly attractive in cancer treatment because micro acidity is a characteristic of TME (Lu et al., 2021). CaCO_3_ can be dissolved into non-toxic products (Ca^2+^, CO_3_^2−^) in some acidic environments, which has been used as a drug delivery system (Zhao et al., 2022b; Chiang et al., 2022). Xu et al. (2022a) loaded anticancer drugs 5-fluorouracil (5-FU) and CUR with CCNM as carriers and placed the drug-loaded CCNM in phosphate buffers (pH 5.8, 6.6, 7.4) to observe the pH-dependent release of drugs. It was found that the release of 5-FU increased with the decrease of pH value, and the results showed that CCNM had good pH control drug release performance. Therefore, CCNM have great potential in acid responsive drug delivery.

### 4.2 Biocompatibility

The biosafety of nanoparticles is crucial in biomedical applications. The reduction in particle size and larger surface area makes them better absorbed by body fluids and tissues, thereby increasing their toxicity (Dong et al., 2022). Unlike other nanoparticles, CCNM have natural biocompatibility because Ca^2+^ and CO_3_^2−^ ions are already widely present in the human body and have not shown cytotoxic effects in normal cell and *in vivo* studies (Yang et al., 2021b). Li et al. (2020b) observed through the use of rabbit red blood cells that CCNM did not cause any cell aggregation or dissolution, indicating that it has good blood compatibility.

### 4.3 High load capacity

The relatively large specific surface area of CCNM gives them high loading capacity. At the same time, the high porosity and well-developed internal structure of CCNM allow for the accommodation of molecules with different properties (Atchuda et al., 2022). These particles can effectively capture various bioactive substances, including low molecular weight compounds and large molecules. These bioactive substances are loaded onto CaCO_3_ through physical adsorption into pores or co precipitation during particle formation, which is very important in delivery systems (Kanwal et al., 2022).

### 4.4 Low cost and easy synthesis

Due to high costs and potential material safety issues, new nano formulations used for targeted drug delivery are significantly hindered when transitioning from the laboratory to the clinical setting. CaCO_3_ is the most abundant mineral in nature, its production cost is significantly lower than that of other nanoparticles, making it a cheap inorganic material (Kaiping et al., 2023). Therefore, CCNM are an extremely important material in the biomedical field. At the same time, compared with other nanomaterials, the preparation of CCNM only requires the use of basic salts (calcium salts and carbonates) and often does not require the use of organic solvents. This simple manufacturing process reduces the cost of CCNM (Trushina et al., 2022).

### 4.5 Easy functionalization

Easy functionalization is also one of the huge advantages of CCNM. Many studies have functionalized it through dry, wet, and *in-situ* modifications to enhance stability and targeting (Han et al., 2019). Due to its small particle size, high specific surface area, and high specific surface energy, CCNM are prone to particle aggregation and agglomeration during preparation and post-treatment, resulting in the formation of secondary particles, increasing particle size and losing the functionality of ultrafine particles during final use, thereby affecting practical application results (Lee et al., 2019; Ippolito et al., 2020). The key to solving this problem lies in the surface modification of CCNM, reducing the adhesion between particles, and improving its dispersibility and dispersion stability in the matrix (Li et al., 2019; Shen et al., 2019). Meanwhile, CCNM still cannot achieve precisely targeted therapy solely relying on passive targeting and acid responsiveness. Therefore, it is possible to endow the delivery system with targeting ability by coupling CCNM to targeted molecules. After the functionalization of CCNM, its *in vivo* stability in the blood will be higher, avoiding macrophage capture and possessing certain targeting characteristics, allowing the carrier to accumulate at specific sites (Dang et al., 2019). Li. et al. (2021d) developed a multifunctional CCNM for the targeted treatment of breast cancer and inhibition of metastasis. The CCNM were functionalized with folic acid molecules (FA) and coated with the T-cell membrane. Within the acidic TME, the CCNM undergo decomposition, leading to the release of Ca^2+^ ions and FA. The liberated FA molecules selectively bind to cancer cells via folate receptors and effectively suppress cancer cell migration, invasion, and proliferation. Notably, the incorporation of T cell membranes imparts tumor-targeting capabilities to the material while also preventing immune clearance within the body. This gives CCNM a prominent advantage in drug delivery systems for cancer treatment.

## 5 The challenges and recommendations for future studies of CCNM

CCNM have been developed, and their biocompatibility, pH responsiveness, and simple preparation have good prospects in the field of tumor treatment. However, the challenges faced in treatment cannot be ignored.

### 5.1 Therapeutic effect of CCNM

In normal individuals, there is also a certain amount of Ca^2+^, so it is difficult to achieve effective therapeutic effects. Moreover, the Ca^2+^ channel/pump on the cell membrane has a strong regulatory function, which makes the cell Ca^2+^ overload quickly return to the normal level, resulting in poor anti-cancer effects (Marchi et al., 2020; Tinawi, 2021). Therefore, combination therapy can further increase the Ca^2+^ content to achieve effective therapeutic concentration. In recent years, drug delivery systems constructed using CCNM biomaterials have been widely studied, but the concentration of drugs delivered is limited and cannot achieve clinically effective concentrations, which cannot achieve the desired clinical effects. Some studies have shown that various molecular adjuvants, such as biopolymers or synthetic polymers, can be added to the CCNM to load more drugs and make the drug release more slowly (Zhou et al., 2023).

### 5.2 Biosafety of CCNM

Ca^2+^ is an element contained in the human body, which has good biocompatibility and biodegradability (Wang et al., 2021). However, when the Ca^2+^ content exceeds the limit, the effect of the Ca^2+^ steady-state regulation system is not very clear. Meanwhile, the inevitable leakage of Ca^2+^ in body fluids is a long-term issue for nanomedicine delivery systems (Bai et al., 2021). Excessive Ca^2+^ can trigger coagulation reactions, leading to the formation of blood clots and posing a threat to organisms (Wang et al., 2021). Moreover, a substantial number of studies solely assess the short-term toxicity of CCNM in mice by delineating organ damage subsequent to CCNM injection, which still poses challenges for accurately evaluating the biosafety of CCNM. So it is imperative to acknowledge the long-term potential risks associated with CCNM. Therefore, in order to facilitate the clinical translation of CCNM, it is imperative to conduct a comprehensive evaluation of the long-term effects of CCNM across various animal models, ranging from rodents to mammals.

### 5.3 Clinical research on CCNM

CCNM have been widely studied in the field of tumor diagnosis and treatment, but there are still some challenges before clinical transformation (Dong et al., 2020). The present preparation processes of CCNM are instable, which easily leads to large particles (Vavaev et al., 2022). Hence, it is imperative to devise precise techniques enabling control over size, composition, and surface modification in order to facilitate large-scale production of CCNM. In addition, the prediction of drug release kinetics for CCNM remain challenging. Despite extensive research on the pH sensitivity of CCNM, their release behavior under normal physiological conditions has not been comprehensively evaluated.

## 6 Conclusion

We have summarized the different roles of CCNM in treating cancer based on the synthesis methods and advantages of CCNM. Among all of the producing methods of CCNM, gas diffusion method is mostly accepted in the medical research field. How to create simpler, more effective methods to produce CCNM with wanted size, crystal forms, and morphologies has been still the research focus as for the drug delivery system in treating cancer; plus, large-scale and controllable industrial production methods to further reduce the cost of CCNM and obtain carriers with higher targeting and drug loading are needed to further explore in the future. It is the characteristics of high loading efficiency and acid responsiveness to TME that making us give more attention to CCNM than other nanomaterial. Actually, Ca^2+^ itself is the universal ion in the cell, functions in nearly all aspects of the living activity, thus, Ca^2+^ overload in certain cells as cancer cells would do no harm to other normal cells if the perfect precision are achieved. On the other hand, CCNM are the drug delivering carrier per se, which can carry whether the chemotherapy agents or novel targeting drugs, even gene editing tools; to some extent, making precise targeting treatment more easier to realize. The most interesting thing is that the Warburg effects happens in cancer metabolism, which acts as the lure for CCNM because of the acid respond property of CCNM, that is, CCNM are not simply as the carrier, it is the metabolic interference in the cancer cells. Based on all these points, CCNM might be the ideal material in fighting the cancers.

Frankly, their low stability in aqueous solutions and insufficient targeting still require researchers’ attention. Moreover, the researchers still need to provide animal models for extensive research to evaluate the long-term effects of CCNM on the living body before clinical conversion.
